# Roles of glycosaminoglycans as regulators of ligand/receptor complexes

**DOI:** 10.1098/rsob.180026

**Published:** 2018-10-03

**Authors:** Robert G. Smock, Rob Meijers

**Affiliations:** European Molecular Biology Laboratory (EMBL), Notkestrasse 85, 22607 Hamburg, Germany

**Keywords:** glycosaminoglycans, protein/protein interactions, drug targets, extracellular matrix, circuit modifiers

## Abstract

Glycosaminoglycans (GAGs) play a widespread role in embryonic development, as deletion of enzymes that contribute to GAG synthesis lead to deficiencies in cell migration and tissue modelling. Despite the biochemical and structural characterization of individual protein/GAG interactions, there is no concept available that links the molecular mechanisms of GAG/protein engagements to tissue development. Here, we focus on the role of GAG polymers in mediating interactions between cell surface receptors and their ligands. We categorize several switches that lead to ligand activation, inhibition, selection and addition, based on recent structural studies of select receptor/ligand complexes. Based on these principles, we propose that individual GAG polymers may affect several receptor pathways in parallel, orchestrating a cellular response to an environmental cue. We believe that it is worthwhile to study the role of GAGs as molecular switches, as this may lead to novel drug candidates to target processes such as angiogenesis, neuroregeneration and tumour metastasis.

## 1. The concept

**Figure 1.**
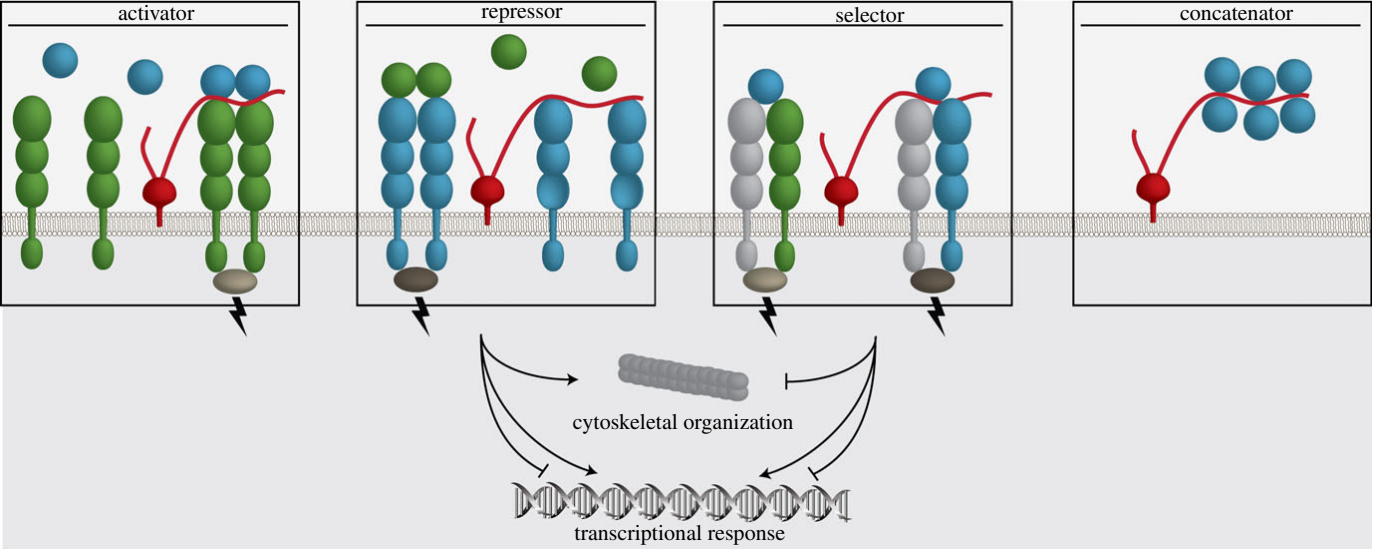
GAGs as extracellular switches in ligand/receptor complex formation. Schematic of GAG polysaccharides (as strings attached to proteoglycans embedded in the membrane, in red) regulating the formation of ligand/receptor complexes. Ligand-induced receptor pairing leads to a signal across the cell membrane that can trigger cytoskeleton reorganization and a transcriptional response. GAG chains act as an activator when the formation of a ligand/receptor complex is facilitated. GAG chains that block the ligand-binding site on a receptor act as a repressor. Combining the function of activator and repressor, GAG chains can favour a certain ligand/receptor complex, acting as a selector. When a GAG chain is of sufficient length, it can act as a concatenator that strings receptors or ligands together at the cell surface.

Glycosaminoglycans (GAGs) are a class of long unbranched polymers of amino and uronic sugars. Many extracellular matrix proteins and receptors bind GAGs, and they are also involved in forming protein–protein complexes [1,2]. GAG molecules are very acidic, and they bind proteins on positive patches often delineated by clusters of arginines and lysines that surround the sugar-associated sulfate groups. When a GAG polymer binds to the positive patch, it neutralizes the protein surface and may facilitate or enhance binding of a protein partner. On the cell surface, the GAG polymer can act as an *activator* that helps to assemble a receptor/ligand complex (figure 1). There are also receptor/ligand complexes that bind through surfaces that have complementary charges. In this case, the GAG polymer binds to the partner that has a positively charged patch, and it may prevent binding of the partner with a negatively charged patch. The GAG polymer acts as a *repressor*, inhibiting formation of this specific complex. In addition, combining the roles described above for repressor and activator, binding of the GAG polymer might favour formation of one complex over another, in which case it acts as a *selector*. There is yet another scenario, where long-chain GAG polymers can string together multiple proteins with positively charged patches. The GAG polymer acts as a *concatenator*, bringing multiple receptor/ligand complexes together on the cell surface (figure 1).

The mediation of interactions between receptor and ligand through GAG polymers is clearly manifold, and can be categorized as individual molecular switches. The sulfation pattern on GAGs can vary extensively, creating an enormous variety that could be exploited by specific ligand/receptor pairs. Binding studies using chips coated with different GAG families have indicated that individual ligand/receptor pairs may interact stronger with certain GAG families [3], but there is also a lot of cross-reactivity. It can, therefore, be assumed that a singular GAG polymer may interact with different ligand/receptor pairs, leading to a coordinated response between different signalling systems. To our knowledge, these connections are underexplored but they may be crucial in understanding how different ligand/receptor systems are linked.

GAG polymers can be released into the extracellular matrix (ECM), creating a local chemical environment that will affect neighbouring cells. In addition, cells can present proteoglycans on the cell surface such as glypicans and syndecans, which carry large GAG polymers attached to the ectodomain. As a migrating cell passes through the ECM created by surrounding tissues, it may encounter a shift in the GAG composition that will affect the receptors present on the migrating cell. Some receptors may become blocked and inhibited, other will be activated, and the cell may also accumulate GAG components and carry them along as they migrate further. A shifting response to the GAG composition in the ECM thus provides a rapid mechanism to influence processes within the migrating cell, a mechanism that is much faster than the endocytosis and exocytosis of receptors. A receptor may be present on the surface of a cell, but it is not responsive until it encounters the right GAG composition. It is striking that many systems involved in tissue remodelling and cell migration are regulated by GAG polymers. Based on recent structural characterizations of these systems, we will describe the different GAG switches that we have so far encountered.

## 2. GAG activators mediate formation of signalling complexes

**Figure 2.**
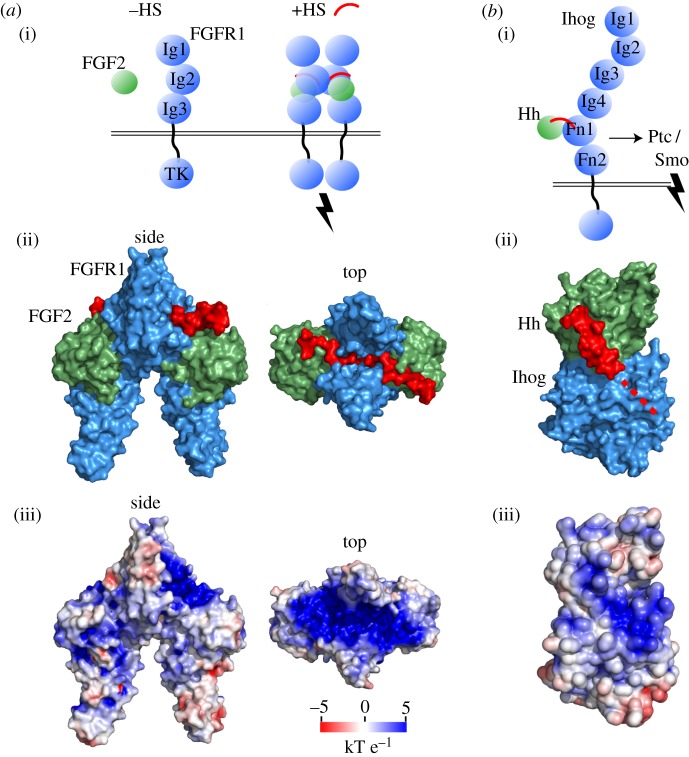
GAG activators. (*a*) (i) Secreted FGF (green) interacts weakly with its receptor FGFR (blue). HS (red) stabilizes the interaction and activates the FGFR dimer for downstream signalling through intracellular tyrosine kinase (TK) domains. (ii) The symmetric complex of two human FGF : two human FGFR (Ig2 and Ig3) : two heparan decasaccharide is shown in a molecular surface representation (PDB: 1FQ9). (ii) The protein electrostatic surface potential in the FGF_2_ : FGFR_2_ assembly shows a contiguous, positively charged surface compatible for HS binding. (*b*) (i) Tighter binding of secreted Hedgehog (green) to insect receptor Ihog (blue) with HS allows stronger interaction with the Patched co-receptor, thereby relieving inhibition of Smoothened for pathway activation. (ii) Crystal structure of fly HhN (green) and domains Fn1–Fn2 of fly co-receptor Ihog solved in complex with HS (PDB: 2IBG). HS is crystallographically unresolved and is partly modelled by superposition with ShhN-HS (PDB: 4C4N), followed by the likely continuation of HS along the dotted line to staple the complex. (iii) Electrostatic surface coloured in the same scaling as in (*a*), with a contiguous positively charged region spanning the Hh–Ihog interface. Electrostatic surface potential was calculated with APBS [11].

Fibroblast growth factors (FGFs) are morphogens that activate signalling involved in angiogenesis and tissue remodelling through binding to FGF receptors (FGFRs) [4]. Heparan sulfates (HSs) play an essential role in these processes [5,6]. Moreover, the heparan sulfate proteoglycan (HSPG) syndecan participates an the internalization and endosomal sorting of FGFR in an FGF-dependent manner [7]. As a biophysical basis for these activities, HS was found to mediate oligomerization of FGF1 [8]. A larger assembly of FGF2, FGFR1 and HS was also observed in a symmetric complex with stoichiometry of 2 : 2 : 2 [9]. HS is required as a scaffold for an even larger (FGF : FGFR)_2_ assembly, such as that of (FGF23 : FGFR1 : α-Klotho)_2_ [10]. Taken together, the (FGF : FGFR)_2_ assembly forms a large and contiguous positively charged groove bridged by HS (figure 2*a*). Accordingly, the most highly sulfated chains of HS have the greatest impact on eliciting FGF2/FGFR-mediated ERK1/2 signalling [12], which may relate to tissue specificity of sulfation patterning in the response to morphogens for both FGF2 [12] and FGF1 [13]. HS is an activator of the molecular assembly of FGF with FGFR and consequent activation of downstream intracellular signalling.

In axon guidance, binding of the guidance cue Slit to the Robo receptor family provides a chemorepulsive signalling mechanism that guides the directional movement of axonal growth cones across the midline. The second leucine-rich repeat domain of the family of Slit proteins contains a relatively flat Arg and Lys-rich C-terminal cap that facilitates HS binding [14–16]. HS forms a ternary complex with Slit-Robo that strengthens the cue–receptor interaction, demonstrating HS activation for chemorepulsive circuit response. Consistent with this scheme, mutations in Slit2 that disrupt HS binding cause loss of the biological activity of chemorepulsive growth cone collapse using axons cultured from *Xenopus* eye primordia [14]. These positively charged residues are conserved [14] and are also implicated in HS binding to Slit3 [2].

Another HS activator is found in the binding of Sonic hedgehog (Shh), which is also involved in the migration of commissural axons across the midline [17]. The Hedgehog (Hh) guidance cue interacts with several receptors, perhaps most centrally with Patched to relieve inhibition of Smoothened for pathway activation [18,19]. Hh forms a complex with Ihog, an insect co-receptor that potentiates pathway activation and allows stronger binding of Hh to Ihog plus Patched than to either Ihog or Patched alone [20]. HS enabled co-crystallization of Hh–Ihog [21]. While HS is not well ordered in this structure, superposition of the Shh–HS crystal complex reveals that the positively charged HS-binding groove in Hh is contiguously extended across the binding interface to Ihog (figure 2*b*) [21,22].

## 3. GAG repressors disrupt signalling complexes

**Figure 3.**
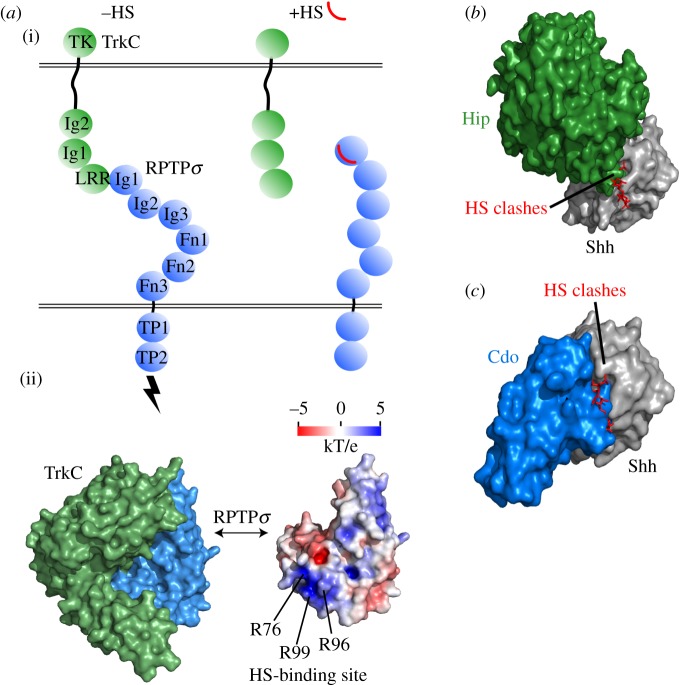
GAG repressors. (*a*) (i) Receptors RPTPσ (blue) and TrkC (green) dimerize and activate downstream signalling through intracellular tyrosine phosphatase (TP) and kinase (TK) domains. Binding of HS (red) to RPTPσ disrupts the interaction with TrkC. (ii) Crystal structure of chicken RPTPσ (Ig1–Ig2) and chicken TrkC (LRR–Ig1) in the absence of HS (PDB: 4PBV). Electrostatic surface potential of RPTPσ is shown in the same orientation. A positively charged surface patch in RPTPσ is buried in the RPTPσ–TrkC interface and includes three Arg residues that have been identified in HS interaction [1,28]. (*b*) In Sonic hedgehog (Shh) signalling, binding of HS to Shh indicates a major steric clash that precludes binding to co-receptor Hip. The crystal structure displayed is of mouse ShhN (grey) in complex with the C-terminal region of human Hip (green) (PDB: 2WFX), solved in the absence of HS. The clash with HS is modelled by superposition with ShhN-HS (PDB: 4C4N). (*c*) A similar HS clash is seen in the same binding hotspot with mouse ShhN (grey) and human co-receptor Cdo domain Fn3 (blue) (PDB: 3D1M).

Receptor protein tyrosine phosphatases (RPTPs) act in the repulsion of axonal growth cones from the midline in embryonic development by interacting with the Slit (guidance cue)/Robo (receptor) signalling system [23], for instance, by binding of *Drosophila* Robo3 and RPTP69d [24]. Moreover, RPTPσ acts in the formation of excitatory synapses [25,26]. One such complex is mediated by receptors RPTPσ and TrkC, which interact across the synaptic cleft between neurons [27]. The structure of the RPTPσ–TrkC binding interaction reveals overlap and binding competition with the RPTPσ–HS interaction [1]. In the RPTPσ–TrkC assembly, HS demonstrates a biophysical basis of a repressor through competitive binding at the RPTPσ molecular surface (figure 3*a*).

Hedgehog interactions also comprise HS repressors. The co-receptor Hip binds Shh at its HS binding site, indicating a major steric clash when both HS and Hip structures are superimposed that is incompatible with both binding simultaneously (figure 3*b*) [22,29]. A similar scenario is observed for the Shh–Cdo complex, whose formation was not observed in the presence of HS (figure 3*c*) [20,22].

## 4. GAG polymers can act as concatenators in signalling, connecting different signalling complexes within the pathway

**Figure 4.**
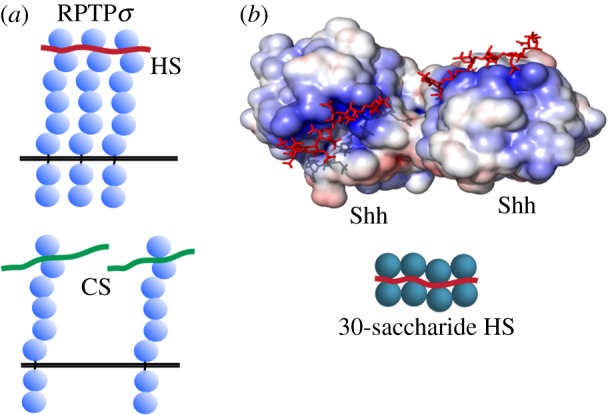
GAG concatenators. (*a*) HS clusters RPTPσ receptors, whereas the alternative chondroitin sulfate GAG of the same chain length inhibits RPTPσ clustering. (*b*) Secreted Shh dimerizes in complex with HS, with some loss of crystallographic resolution in the intermediate HS chain. The N-terminal signalling domain of mouse ShhN is coloured by electrostatic surface potential (PDB: 4C4N). Incubation with longer HS polysaccharides indicates the stacking of higher-order Shh multimers along the HS chain.

Remarkably, HS and chondroitin sulfate (CS) are both ligands for RPTPσ and have opposing effects on RPTPσ behaviour [28,30,31]. HS oligomerizes RPTPσ in solution and HSPGs colocalize with RPTPσ on sensory neurons and promote their extension. Whereas HS acts as a concatenator, CS that has a comparable chain length inhibits RPTPσ oligomerization, and inhibits neuronal extension (figure 4*a*) [28].

GAG interaction within the Shh pathway also illustrates how GAG can regulate Hh function as a concatenator. A recent crystal structure of Shh reveals a characteristic Arg- and Lys-rich surface patch that binds HS and CS, and, given sufficient chain length such as found in the HSPG glypican-3, enables multimeric assembly of Shh along the HS polymer (figure 4*b*) [22]. As in most Hh structures, calcium and zinc cations shield potentially repulsive interactions between acidic residues and HS and invert the surface electrostatic potential at these sites.

## 5. A candidate GAG switch for netrin receptor selection in axon guidance

**Figure 5.**
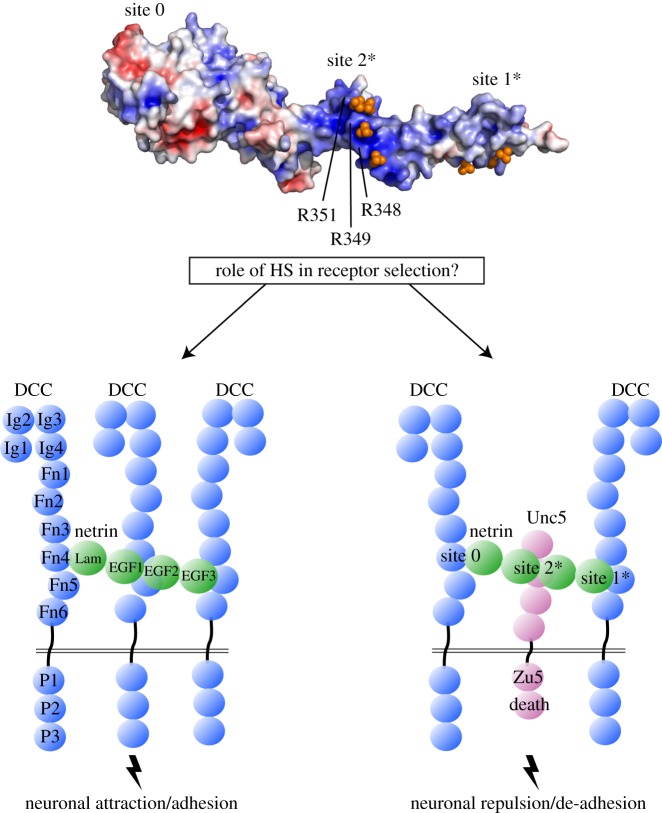
GAG selector. HS may modulate the selection of netrin receptors in axon guidance signalling. Human netrin-1 (PDB: 4URT) is shown with electrostatic surface potential and three labelled binding sites for the receptors DCC and Unc5 [39–41]. Positive surface charge clusters (blue patches) and series of crystallographic sulfate ions (orange spheres) suggest putative HS binding at sites 1 and 2 (marked with *). Moreover, DCC and Unc5 appear to be in binding competition at an Arg-rich, sulfate-binding region of netrin site 2, suggesting a model in which HS acts in netrin receptor selection and axon guidance fates.

The guidance cue netrin acts as an attractant [32] or repellent for axonal growth cones [33], and its depletion has been linked to apoptosis [34]. How the same guidance molecule can trigger such diverse cellular responses has been under intense investigation. Netrin can bind to a diverse set of cell surface receptors, including DCC [35], UNC5 [33], DSCAM [36], amyloid precursor protein [37] and Cd146, which is involved in angiogenesis [38]. Netrin triggers different signals depending on the receptors present on the cell surface. This has been observed in the turning responses of axons that either contain DCC alone (causing chemoattraction) [35], or both DCC and UNC5 (causing chemorepulsion) [33] (figure 5).

Netrin function has also been associated with HS. In particular, HS affects DCC function as an attractant [42] and forms a complex with netrin and DCC [43]. Recently, it has been shown that a glypican HSPG (Lon2) functions in axon guidance through netrin signalling in *Caenorhabditis elegans* [44]. A microarray containing different forms of HS further indicates that netrin favours the binding of some HS varieties over others [45], suggesting that there may be specific interactions between netrin and HS that affect netrin signalling.

Multiple binding sites between netrin and DCC demonstrate mechanisms of netrin-mediated DCC homodimerization [39,40]. Based on site-specific mutants of netrin and their influence on axonal turning in the presence of DCC alone or in combination with UNC5, it was proposed that DCC and UNC5 share a common binding site on netrin-1 that lies between domains EGF-1 and EGF-2 [40]. UNC5 and DCC would compete for binding at this site, allowing a DCC/DCC homodimer or a UNC5/DCC heterodimer to form (figure 5). The UNC5 binding site on netrin-1 was confirmed by complementary biophysical studies using domain deletions and mutants [41]. The most important residues on netrin that determine both DCC and UNC5 binding include a cluster of five conserved arginines, and mutation of two of them (Arg349 and Arg351) abolishes binding of both DCC and UNC5 [40,41]. Interestingly, these arginines bind to four sulfate ions in the crystal structure of the netrin–DCC_FN5FN6_ complex [40]. The cluster of sulfate is at such a close range that it resembles a unit of HS. Indeed, HS can be fitted using the sulfate ion cluster as a guide. It therefore seems likely that HS acts as a switch that alters the binding competition of DCC and UNC5 for netrin and modifies the circuit of axonal attraction and repulsion. However, a precise mechanistic role has not yet been depicted of HS as a switch for netrin receptor selection, and is a promising area of future investigation.

## 6. GAG circuit complexity, crosstalk and regulation in a broader physiological context

Data mining has uncovered at least 435 human proteins that interact with HS alone. The most prominent enrichment of gene ontology terms are found in cell–cell signalling, development, cell proliferation and immunoresponse [46], which are dependent on complex signal processing behaviour from a mixture of external cues. HSPGs in the membrane-bound extracellular domain of cells are increasingly appreciated for their role in signalling mechanisms and axon guidance of diverse organisms [47]. Modulation of cue–receptor engagements by HS, and restructuring of the sulfation pattern on the extracellular surface [48], suggest clues for how cells respond to the appropriate signals in a complex milieu of extracellular binding partners. Different GAGs may favour different cue/receptor engagements, and the presence of a particular GAG in a confined area may amplify the effect of specific cues and thus regulate certain migration patterns. The so-called sugar code for axon guidance may thus be linked to the regulation of cue/receptor complexes [49–51].

In Shh signalling, the Patched receptor also binds to Shh overlapping the same molecular interface as HS, Hip and Cdo [20,52,53], which may, in part, explain the competition among these proteins for Shh and signalling attenuation by mammalian HSPG Gpc-3 [54]. The HS-binding Arg/Lys surface residues in Shh serve as a crucial site with elaborate HS switching circuitry. Mutation of these residues leads to phenotypes associated with defective binding to HS, reduced Shh multimers, defective binding to Patched, weak signalling and downstream gene transcription activity, and developmental disorder [55–59]. Breaking the myriad interactions by mutation of the HS-binding hotspot on Hedgehog seems difficult to associate with any single causal factor, underscoring the perspective of a GAG-controlled integrated circuit.

**Figure 6.**
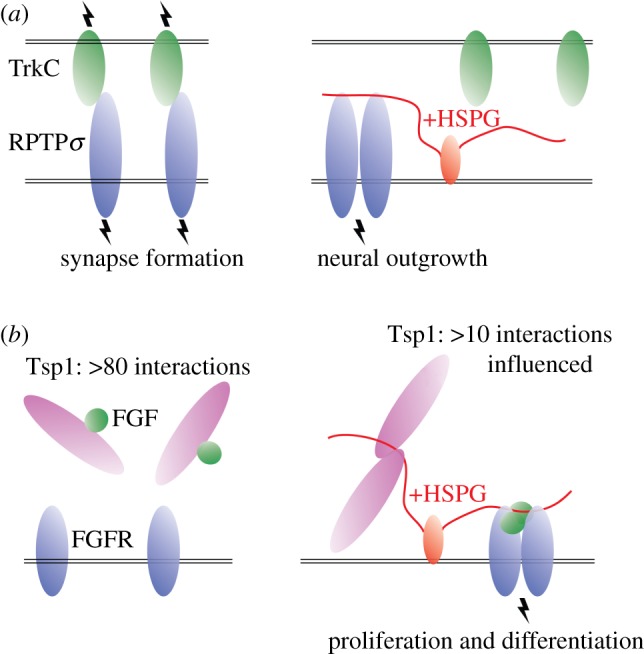
Glimpses of crosstalk in broader physiological context. (*a*) In the absence of HS, RPTPσ favours *trans*-synaptic binding and synaptic signalling with TrkC. In the presence of HS, RPTPσ–TrkC interaction is disrupted and RPTPσ clustering and neuronal outgrowth are promoted. (*b*) The extracellular matrix protein Tsp1 binds a variety of proteins and receptors in the absence of HS, including FGF. HS mediates Tsp1 clustering and other Tsp1 interactions, including release of FGF from Tsp1 and formation of the FGF–FGFR signalling complex.

Furthermore, the same biophysical mechanisms of GAG repression and activation in signalling are also observed in a variety of other extracellular interactions: for example, *trans*-cellular receptor binding in synapse formation and in interactions among extracellular matrix proteins. HS, but not CS, concatenates RPTPσ, while HS represses RPTPσ and TrkC interaction. HS seems to provide a contextual switch between different biological activities in neuronal extension (RPTPσ clustering; HS concatenator) and stable synaptic formation (RPTPσ–TrkC; HS repressor) (figure 6*a*).

Larger, more complex circuits and elaborate signal processing behaviour may also emerge from a more complete understanding of extracellular protein interactomes and their modulation by GAGs. Thrombospondin-1 (Tsp-1) is an extracellular protein that binds a wide array of matrix proteins, guidance cues, receptors and proteases. Of more than 80 interaction partners, at least 10 are influenced by HS [60]. Accordingly, Tsp-1 binds the HSPG co-receptors syndecan and glypican [61] in addition to extracellular matrix CSPGs aggrecan and versican [62]. Many of the Tsp-1 interactions are inhibited by HS, suggesting that HS provides control over the linkages among membrane and extracellular components [60]. For instance, binding of HS blocks the interaction of Tsp-1 with bFGF (FGF2) [63]. Combined with the scheme of HS-mediated assembly of FGF2 with FGFR-1 (HS activator in figure 2*a*), and the myriad of interactions mediated with Tsp-1 that are also influenced by HS, Tsp-1 appears to provide a mechanistic platform for context-dependent crosstalk among signalling systems (figure 6*b*). The biophysical basis of interaction on Tsp-1 is through HS binding to the Tsp-1 N-terminal laminin G-like domain in an arginine-rich surface patch [64]. Furthermore, HS homodimerizes Tsp-1 G-like domains in alternate configurations, suggesting orientational plasticity among HS-mediated Tsp-1 interactions [65].

The presentation of HSPGs in extracellular communication networks involves regulated expression of HSPGs themselves and extracellular enzymes that modify GAGs, either by cleaving a GAG from its core protein or altering its pattern of sulfation. Knockout studies on biosynthetic enzymes involved in GAG production in *C. elegans* have shown a strong effect on the migration behaviours of neurons [66] and their axons [67]. A marked difference in the natural expression of an HS-degrading enzyme, HPSE, was observed in differentiating versus proliferating human olfactory epithelium cells [68], and HPSE has been found to alter Shh and Wnt signalling in human medulloblastoma cells [69]. RNAi-mediated knockdown of extracellular enzymes that modulate HS sulfation patterning, Sulf1 (removes sulfate from HS) and Hs6st (transfers sulfate to HS), have opposing effects on neurotransmission. They result in misregulation of HSPGs such as glypican (Dlp) and syndecan, high abundance of the guidance cues Wnt and Bmp, and impaired endosomal cycling with the Wnt receptor Frizzled [70,71] in a process that is essential for Wnt signalling [72,73]. HSPGs assemble lipidated, multimeric Shh on the surface of Hh-producing cells [74] and recruit Scube2 for Shh processing and shedding in a manner that is both HS sulfation-dependent and cell-dependent [75]. Understanding the biological reality of systems-level GAG circuits is further challenged by the finding that not all HSPGs behave equally. Hedgehog and Wnt signalling regimes may be either stimulatory or inhibitory in relation to co-expression of a variety of different HSPGs, indicating HSPG specialization that also involves contribution of the protein core [76,77].

## 7. Evolutionary selection of GAG switch variants

**Figure 7.**
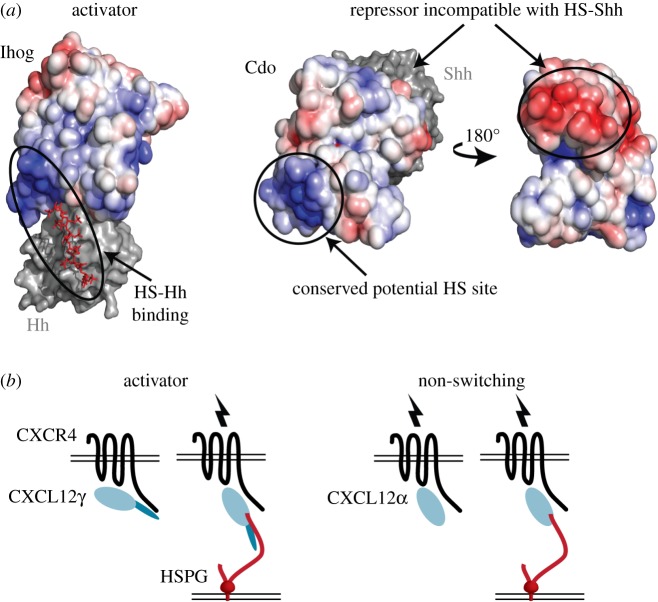
Evolved variations of GAG switches. (*a*) In hedgehog signalling, the mammalian Cdo receptor shares common ancestry with insect Ihog. However, the hedgehog-binding site is not conserved and indicates a reversal in compatibility for simultaneous binding with HS. A composite model of Cdo domains Fn2 (PDB: 1X4Z) and Fn3 bound to Shh (PDB: 3D1M) was created by superposition with the most closely homologous domains of Ihog bound to Hh (Fn1–Fn2, PDB: 2IBG) and coloured by electrostatic surface potential. Comparison to the same orientation of Ihog shows conservation of a positively charged surface patch used for HS binding (blue), while hedgehog binds to a different domain of Cdo at a negatively charged surface patch (red) in the absence of HS. (*b*) Chemokine isoforms CXL12γ/α stimulate signalling with the GPCR CXCR4. However, CXL12γ contains an auto-inhibitory tail that requires HS binding for activation, while HS is not required for stimulation by CXL12α. Model adapted from Coles *et al*. [28].

The evolution of the role of GAG switch variants within a ligand/receptor protein family further emphasizes how they can adapt the receptor signalling response to the environment. In the Hh signalling pathway, HS acts as an activator for interaction of insect Ihog and Hh (figure 2*b*). By contrast, HS serves as a repressor for mammalian Cdo with Shh (figure 3*c*). Remarkably, the relevant binding regions of these receptors (Ihog Fn1–Fn2 and Cdo Fn2–Fn3) share common ancestry but diverge in HS circuit modification [20]. Cdo is an HS-binding protein and retains the positively charged HS binding site of Ihog (figure 7*a*) [78]. However, while Cdo retains the HS binding site in Fn2, its role in mediating interaction is not yet clear, and Cdo has separated its HS and Hh binding sites to different domains. In a mode of Hh binding that is distinct from Ihog, the adjacent Fn3 domain of Cdo binds Shh via the Shh–HS binding site. Assembly of Shh–Cdo is stable in the absence of HS and is structurally incompatible with HS [20]. Therefore, the role of HS in Hh–Ihog/Cdo interaction has undergone evolutionary divergence as an activator in insects and a repressor in mammals.

Evolutionary selection among other GAG switch variants has also been observed. Chemoattraction in the migration of leucocytes is mediated by the binding of CXCL chemokine isoforms to the GPCR receptor CXCR4. The CXCLγ isoform contains a C-terminal-motif rich in Lys residues that binds sulfated tyrosine residues of CXCR4 and acts as an inhibitory element that does not result in chemotactic signalling [79]. In the presence of HS, CXC12γ remains bound to CXCR4 and activates signalling, apparently in a conformation that relieves C-terminal inhibition (figure 7*b*). Alternatively, the CXCL12α isoform has a C-terminal truncation of the Lys-rich region and activates the CXCR4 receptor without requirement for HS to relieve inhibition [79]. The evolutionary selection of isoforms with simple genetic adjustment provide for both HS-dependent (activator) and HS-independent (non-switching) circuits. A related scenario is seen in the HS-dependent *trans*-synaptic clustering of the HSPG Gpc4 with LRRTM4, but not with its isoform LRRTM2 [80].

## 8. The potential of GAGs as drug targets

The capacity of sulfated moieties such as HS to function as switches of biological interaction raises their potential for therapeutic intervention. An important question that has to be answered in the future is whether the GAG interactions are specific for particular receptor/ligand complexes. If these interactions are generic, many processes will be linked by a common GAG pool and it will be difficult to identify a particular GAG as a drug target. However, there are several indications that this is not the case, and that GAG specificity is important. The knockout of certain GAG-modifying enzymes has an effect on specific signalling elements [69], indicating that particular modifications of GAGs will affect only certain signalling pathways. There is also a GAG derivative drug on the market that acts as an anticoagulant with relatively small side effects. Fondaparinux is a pentasaccharide that selectively inhibits a serine endopeptidase Xa [81]. Although it is similar in structure to HS, fondaparinux does not seem to affect all GAG-related pathways. Identification of specific GAGs targeting a particular signalling pathway could, therefore, lead to the development of small molecule drugs that affect protein–protein interactions. Since most of these GAG molecules are biocompatible, toxicity will not be an issue even at high doses. Heparin has been used to treat preeclampsia for decades. A cocktail of low-molecular-weight GAGs may not have been very effective [82], but it showed few side effects. Detailed investigations into the relation between GAG structure and its interactions with specific ligand/receptor complexes will benefit drug discovery and may also reveal which components of signalling pathways are linked by the use of specific GAGs

## 9. Concluding remarks

It has become evident that GAGs play a central role in tissue development, yet it remains a challenge to decipher the GAG code. *In vivo* investigation of the role of certain GAGs through the knockout of GAG synthesizing enzymes shows many developmental effects, yet lacks mechanistic clarity. Conversely, reductionist experiments of specific GAG complexes provide detailed mechanistic insights but may not be able to recapitulate full biological consequence. Extracellular signalling may be modelled systematically as directional networks and circuits that integrate such codependencies [48,83]. Extending the metaphor of circuitry to a realistic, mechanistic model in cell guidance is appealing because it has the potential to explain complex signal processing behaviour from discrete, modular parts that can be verified by the reductionist experimental investigation of molecular structure and binding modes [84]. Genetic circuit models comprising logic gates, such as those representing transcriptional activators and repressors, recapitulate downstream transcriptional regimes with altered cell behaviour [85,86]. Accordingly, the representation of GAG circuit motifs as extracellular activators and repressors of specific cue–receptor engagements in the ‘front line’ of environmental sensing may expand on an understanding of signal integration. Moreover, the capacity of truncated GAG chains to function as extracellular switches holds great promise for therapeutic intervention in neural repair and tumour metastasis. A better understanding of the signalling modules affected by GAGs may inform a more sophisticated design of GAG-derived drug candidates.

## Data accessibility

This article has no additional data.
